# Octanoate is differentially metabolized in liver and muscle and fails to rescue cardiomyopathy in CPT2 deficiency

**DOI:** 10.1016/j.jlr.2021.100069

**Published:** 2021-03-20

**Authors:** Andrea S. Pereyra, Kate L. Harris, Arvin H. Soepriatna, Quin A. Waterbury, Sivakama S. Bharathi, Yuxun Zhang, Kelsey H. Fisher-Wellman, Craig J. Goergen, Eric S. Goetzman, Jessica M. Ellis

**Affiliations:** 1 Brody School of Medicine at East Carolina University, Department of Physiology, and East Carolina Diabetes and Obesity Institute, Greenville, NC, USA; 2 Department of Biochemistry, Purdue University, West Lafayette, IN, USA; 3 Weldon School of Biomedical Engineering, Purdue University, West Lafayette, IN, USA; 4 Department of Pediatrics, Children's Hospital of Pittsburgh of the University of Pittsburgh Medical Center, University of Pittsburgh School of Medicine, Pittsburgh, PA, USA

**Keywords:** medium-chain fatty acids, carnitine shuttle, fatty acid oxidation, mitochondria, carnitine palmitoyltransferase, ACSM3, medium-chain acyl-CoA synthetase 3, CACT, carnitine-acylcarnitine translocase, CD, control diet, CPT1, carnitine palmitoyltransferase 1, CPT2, carnitine palmitoyltransferase 2, CrOT, carnitine O-octanoyltransferase, LCAC, long-chain acylcarnitine, LCKD, long-chain fatty acid ketogenic diet, MCAD, medium-chain acyl-CoA dehydrogenase, mFAO, mitochondrial fatty acid oxidation, mTOR, mechanistic target of rapamycin, OctD, octanoate diet, PC, phosphatidylcholine, PDH, pyruvate dehydrogenase

## Abstract

Long-chain fatty acid oxidation is frequently impaired in primary and systemic metabolic diseases affecting the heart; thus, therapeutically increasing reliance on normally minor energetic substrates, such as ketones and medium-chain fatty acids, could benefit cardiac health. However, the molecular fundamentals of this therapy are not fully known. Here, we explored the ability of octanoate, an eight-carbon medium-chain fatty acid known as an unregulated mitochondrial energetic substrate, to ameliorate cardiac hypertrophy in long-chain fatty acid oxidation-deficient hearts because of carnitine palmitoyltransferase 2 deletion (*Cpt2*^*M−/−*^). CPT2 converts acylcarnitines to acyl-CoAs in the mitochondrial matrix for oxidative bioenergetic metabolism. In Cpt2^M*−*/*−*^ mice, high octanoate-ketogenic diet failed to alleviate myocardial hypertrophy, dysfunction, and acylcarnitine accumulation suggesting that this alternative substrate is not sufficiently compensatory for energy provision. Aligning this outcome, we identified a major metabolic distinction between muscles and liver, wherein heart and skeletal muscle mitochondria were unable to oxidize free octanoate, but liver was able to oxidize free octanoate. Liver mitochondria, but not heart or muscle, highly expressed medium-chain acyl-CoA synthetases, potentially enabling octanoate activation for oxidation and circumventing acylcarnitine shuttling. Conversely, octanoylcarnitine was oxidized by liver, skeletal muscle, and heart, with rates in heart 4-fold greater than liver and, in muscles, was not dependent upon CPT2. Together, these data suggest that dietary octanoate cannot rescue CPT2-deficient cardiac disease. These data also suggest the existence of tissue-specific mechanisms for octanoate oxidative metabolism, with liver being independent of free carnitine availability, whereas cardiac and skeletal muscles depend on carnitine but not on CPT2.

Because the heart is a continuously working muscle, it has a high bioenergetic demand and relies primarily on fatty acids as substrate. Fatty acids are particularly important cardiac substrates as indicated by dysregulated mitochondrial fatty acid oxidation (mFAO) in metabolic diseases, such as diabetes, obesity, hypertension, and cardiovascular disease. As such, cardiac energy insufficiency continues to be recognized as the underlying etiology of clinical hypertrophic cardiomyopathy (1). Cardiac hypertrophic remodeling is an independent predictor of all-cause death (2, 3) and a common occurrence in obese (4, 5), hypertensive (6), diabetic (7), and aged (8) individuals, and it is well established that it occurs as a direct consequence of impaired oxidative metabolism in the myocardium. In agreement, animal models of cardiac hypertrophy have documented energetic deficits and/or disruptions in mFAO (9, 10, 11). Moreover, cardiac hypertrophy occurs as a direct consequence of primary defects in mFAO (12, 13, 14, 15, 16). The metabolic pathway of beta-oxidation of long-chain fatty acids for energy is regulated by the transport of fatty acids into the mitochondrial matrix via the carnitine shuttle. The first and required step in mitochondrial long-chain fatty acid oxidation is the activation by acyl-CoA synthetase, being ACSL1 the predominant activator for oxidative metabolism in the heart (13, 17, 18, 19, 20). Once the acyl-CoA moiety is formed, the CoA group is exchanged for a carnitine molecule by the rate-limiting enzyme carnitine palmitoyltransferase 1 (CPT1) to generate an acylcarnitine. The acylcarnitine is then transported through the inner mitochondrial membrane by the carnitine-acylcarnitine translocase (CACT). Once the acylcarnitine is inside the mitochondria, carnitine palmitoyltransferase 2 (CPT2) exchanges the carnitine group for a mitochondrial CoA to recreate an acyl-CoA for subsequent beta-oxidation.

In addition to mFAO, the high-energy demands of the heart are also met by glucose oxidation. Indeed, the loss of cardiac glucose oxidation because of deletion of the mitochondria pyruvate carrier subunits results in cardiac hypertrophy (21, 22). Remarkably, this severe cardiac hypertrophy is rescuable by high-fat or ketogenic medium-chain fatty acid diets (21, 22) suggesting that ketones, long-chain fatty acids, and medium-chain fatty acids could serve as critical alternative substrates for cardiac oxidative metabolism. We and others have demonstrated that loss of myocardial mFAO by the genetic deletion of CPT2 or CPT1 in mouse myocardium results in cardiac remodeling, hypertrophy, and heart failure (14, 23). In patients with severe genetic defects of Cpt2, cardiomyopathy is common, and medium-chain fatty acid-enriched diet is used as therapy. Medium-chain fatty acids of eight carbons or fewer provide an alternative energetic substrate because they are thought to bypass the carnitine-dependent CPT1-CACT-CPT2 shuttling system to access the mitochondrial matrix for subsequent oxidation. Here, we tested the ability of the medium-chain fatty acid octanoate to improve CPT2 deficiency-induced cardiac hypertrophy. Surprisingly, a heavily octanoate-enriched ketotic diet was not able to alleviate CPT2 deficiency-mediated cardiac hypertrophy. This effect is potentially mediated by limited access of the heart to dietary octanoate, as the liver receives the first pass of exogenous octanoate through the portal vein upon intestinal absorption. Here, we found that liver has an additional metabolic advantage because liver mitochondria were able to oxidize free octanoate, whereas the heart and skeletal muscle were not. On the contrary, the carnitine-bound form of octanoate was readily oxidized by heart, skeletal muscle, and liver mitochondria evidencing that the specific octanoate metabolites are oxidized distinctly in a tissue-dependent manner. Despite the requirement of carnitine for octanoate oxidation in muscles, octanoylcarnitine oxidation rates are not impacted by CPT2 deficiency. These data suggest that free octanoate can be directly oxidized in liver but not in cardiac and skeletal muscles. Furthermore, in muscles, octanoate oxidation is carnitine dependent but CPT2 independent. Our data also suggest that mFAO is obligatory for maintaining cardiac size and function.

## Materials and methods

### Animal

Heart- and muscle- (*Cpt2*^*M−/−*^) and skeletal muscle-specific (*Cpt2*^*Sk−/−*^) CPT2 conditionally deficient mice were generated using Cre recombinase driven by the muscle creatine kinase promoter (Jackson Laboratories; stock no. 006475) or the human alpha-skeletal actin promoter (The Jackson Laboratory; stock no. 006149) as described (23, 24). Littermates lacking the *Cre* transgene were used as controls. Mice were given free access to water and standard chow (PicoLab 5053; Lab Diets), in pathogen-free housing under 12-h light-dark cycles. All procedures were approved by the Purdue Animal Care and Use Committee (assurance A3231-01) and the Institutional Animal Care and Use Committee of East Carolina University (assurance A3469-01). For diet studies, mice were given ad libitum access to either control (PicoLab 5053; Lab Diets, Richmond, IN; TD94045; Envigo Teklad, Madison, WI), ketogenic diet (F6689; fat:protein + carbohydrate, 4:1; BioServ, Flemington, NJ), or trioctanoin-enriched diet (TD170585; Envigo Teklad). Trioctanoin (Captex 8000) obtained from Abitec was used for customized diets synthesized by Envigo (supplemental Table S1). All dietary interventions began between week 3 and 6 of age and lasted 4 weeks. For carnitine studies, powdered l-carnitine (Sigma C0283) was dissolved in the drinking water at 300 mg/kg/day, as described (24, 25). Echocardiograms were collected as previously described (23) from isoflurane-anesthetized mice (1–3% in 1.5 l/min medical-grade air) via high-frequency ultrasound (Vevo2100; FUJIFILM VisualSonics). M-mode, B-mode, and respiratory- and cardiac-gated images (electrocardiographically gated kilohertz visualization) were collected for both medial short- and long-axis views (MS550D transducer; FUJIFILM VisualSonics). Echocardiogram-derived calculations were determined from the Endocardial and Epicardial Area Protocol (Vevo Lab; FUJIFILM VisualSonics). Beta-hydroxybutyrate was measured using a commercially available kit according to manufacturer's instruction (StanBio).

### Lipid analysis

Lipid profiling of tissue and biological fluids was performed as previously described (23). Briefly, lipids were extracted from tissues using Bligh and Dyer method (26), and both the lipid and polar phases were dried separately, resuspended, and injected directly via a microautosampler (G1377A) into a QQQ6410 triple quadrupole mass spectrometer (Agilent Technologies, San Jose, CA) operated in the positive-ion mode and equipped with ESI ion source, as we have described (23, 27). Data were collected in multiple reaction monitoring mode (23, 27, 28), and ion intensities acquired by an in-house script were further normalized to sample protein concentration.

### Molecular response and indicators

RNA was isolated using Trizol (Invitrogen), and RNA was converted to complementary DNA (Applied Biosystems High Capacity cDNA RT Kit) and used for SYBR Green (Bio-Rad)-based real-time PCR. Results were normalized to housekeeping gene and expressed as arbitrary units of 2^−ΔCT^. For Western blots, lysates were collected in lysis buffer (50 mM Tris-HCl, 150 mM NaCl, 1 mM EDTA, and 1% Triton X-100) with protease and phosphatase inhibitors. Homogenates, lysates, or mitochondrial pellets were equally loaded and electrophoresed on SDS-polyacrylamide gels, transferred to nitrocellulose membrane, blocked with 5% milk-Tris-buffered saline with 0.1% Tween® 20 detergent for 1 h, incubated with primary antibody (1:1,000–1:2,000) against alpha-tubulin (Sigma T0198); pyruvate dehydrogenase (PDH) and phospho-PDH (S293) (Cell Signaling 3205 and 31866); medium-chain acyl-CoA synthetase 3 (ACSM3; Invitrogen PA5-100374); alpha-AMP-activated protein kinase and phospho-AMP-activated protein kinase (T172) (Cell Signaling 5831 and 2535), washed, and incubated with secondary antibodies conjugated to infrared dye 800CW or 680LT (LiCor). Proteins were visualized with Odyssey and quantified using Image Studio (LiCor). Carnitine O-octanoyltransferase (CrOT) expression in isolated mitochondria from mouse liver and heart was determined by label-free proteomics via nano LC-MS/MS as described previously (29).

### Oxidation assays

Oroboros high-resolution respirometry was performed on fresh whole-tissue homogenate and/or isolated mitochondria from wild-type C57Bl/6J mouse liver, heart, and skeletal muscle and *Cpt2*^*Sk−/−*^ skeletal muscle. Homogenization was performed in Mir05 buffer (MgCl_2_-6H_2_O 3 mM, K^+^MES 105 mM, taurine 20 mM, KH_2_PO_4_ 10 mM, Hepes 20 mM, d-sucrose 110 mM, and fatty acid-free BSA 1 g/l) at 20 or 40 w/v. Mitochondria were isolated by standard differential centrifugation, and yield was assessed by measuring protein concentration in mitochondrial pellets (30). Respiration was measured in Mir05 media (31) using 60 μl of tissue homogenate, 100 μg (liver) or 50 μg (heart and skeletal muscle) of isolated mitochondria in each chamber. After baseline respiration was established, malate (2 mM; Sigma M1296) was added to maintain the tricarboxylic acid cycle, followed by ADP (2 mM) to stimulate respiration. Substrates were added in the following concentrations: free octanoate 0.2 mM (Sigma C5038), octanoyl-CoA 0.2 mM (Sigma O6877), octanoylcarnitine 0.2 mM (Sigma 50892), palmitic acid 0.02 mM (Sigma P0500), palmitoyl-CoA 0.02 mM (Sigma P9716), palmitoylcarnitine 0.02 mM (Sigma P1645), free CoA 0.125 mM (Sigma C3019), and l-carnitine HCl 5 mM (Sigma C0283). Complementary assays were performed in mitochondria isolated in KCl 100 mM, Mops 50 mM, EGTA 1 mM, MgSO_4_ 5 mM, BSA 0.2%, buffer, pH 7.1, and respiration measured in ATP containing buffer (K-MES 105 mM, KCl 30 mM, KH_2_PO_4_ 10 mM, MgCl_2_ 5 mM, EGTA 1 mM, BSA 2.5 g/l, and pH 7.1). The experiments were repeated at least three times with mitochondria isolated from separate animals. To combine data from the three experiments, we calculated the ratio of ADP-stimulated fatty acid-induced respiration rates (lipid + malate in the presence of ADP) to that with malate alone (malate + ADP).

### Medium-chain acyl-CoA dehydrogenase enzyme activity assays

Heart and liver lysates from C57Bl/6 mice were prepared in phosphate-buffered saline supplemented with 0.1% lubrol detergent. Medium-chain acyl-CoA dehydrogenase (MCAD) activity was determined using the electron-transferring flavoprotein fluorescence-based microplate assay exactly as described (32), using 25 μM octanoyl-CoA as substrate.

### Statistics

Data are presented as mean ± SEM, unless otherwise specified. Statistical analysis and figures were generated using Excel or GraphPad Prism, version 8.0.0 for Windows (GraphPad Software). Data were compared using unpaired Student's *t*-test and 1-way or 2-way ANOVA followed by multiple comparison analysis. Significance level was set at *P* < 0.05. Statistical details for each data set are described on figure legends. Heatmapper online tool was used to generate heat maps (Wishart Research Group).

## Results

### Dietary octanoate does not attenuate cardiac hypertrophy induced by CPT2 deficiency

**Fig. 1:**
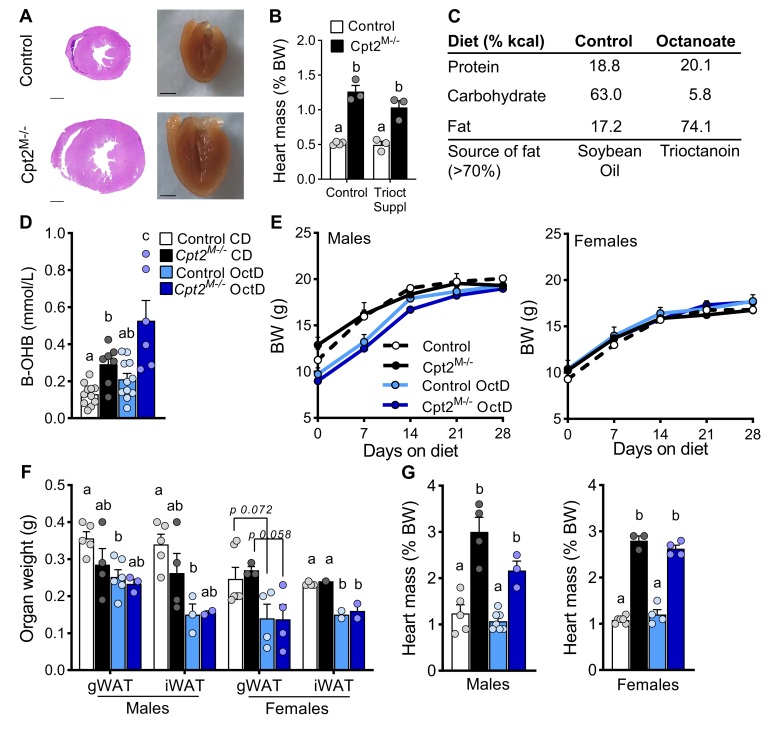
Dietary octanoate failed to attenuate cardiac hypertrophy in *Cpt2*^*M−/−*^ mice. A: Transverse, H&E-stained sections of control and *Cpt2*^*M−/−*^ hearts (left) and photography of half heart post fixative (right). B: Heart weight of control and *Cpt2*^*M−/−*^ mice in response to 20% medium-chain supplemented chow diet. C: Diet composition of formulated control (CD) and octanoate (OctD) diets. D: Plasma ketones in response to 4 weeks on diets, n = 6–9. E: Body, (F) organ, and (G) heart weight of male and female control and *Cpt2*^*M−/−*^ mice fed control or OctD, n = 4–6. Data are presented as mean ± SEM. Statistical analysis by 2-way ANOVA. Means depicting a different letter indicate significant differences between groups (*P* ≤ 0.05). The scale bar represents 1,000 μm. B-OHB, beta-hydroxybutyrate; BW, body weight; Cpt2, carnitine palmitoyltransferase 2.

**Fig. 2:**
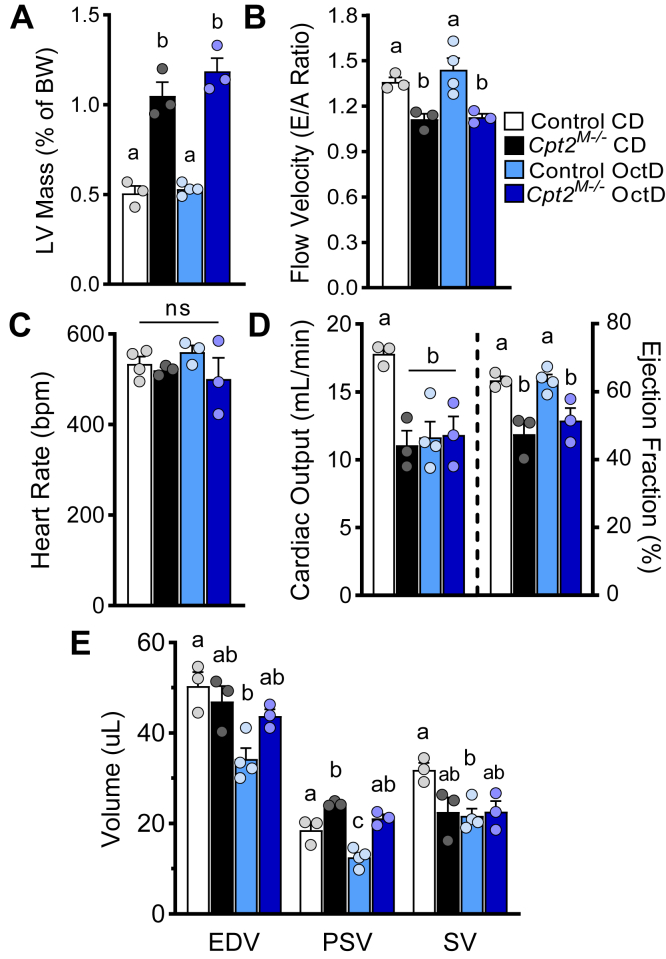
Dietary octanoate did not improve heart function in *Cpt2*^*M−/−*^ mice and increased cardiac output in control mice. A: Left ventricular mass relative to body weight, (B) flow velocity, and (C–E) cardiac functional parameters derived from 2D echocardiogram and Doppler analysis of control and *Cpt2*^*M−/−*^ mice on control or OctD, females, n = 3–4. Data are presented as mean ± SEM. Statistical analysis by two-way ANOVA. Means depicting a different letter indicate significant differences between groups (*P* ≤ 0.05). Cpt2, carnitine palmitoyltransferase 2; EDV, end-diastolic volume; PSV, peak-systolic volume; SV, stroke volume.

Individuals with inborn errors in mFAO, such as CPT2 deficiency, commonly supplement their diets with medium-chain fatty acids as an alternative fuel source. To determine if supplementation with octanoate would alleviate cardiac hypertrophy in CPT2 deficiency (Fig. 1A) by bypassing the carnitine-mediated transport shuttle for mitochondrial oxidation, we fed control and myocardial CPT2-deficient mice (*Cpt2*^*M−/−*^), a diet supplemented with trioctanoin, a triacylglycerol containing three 8-carbon-long acyl chains, as 20% wet weight in the regular diet. However, the octanoate-supplemented diet failed to rescue cardiac hypertrophy (Fig. 1B). Octanoate boluses and medium-chain ketogenic diets can increase circulating ketone bodies (33) because of stimulation of high rates of medium-chain mFAO flux in the liver. Importantly, medium-chain ketogenic diets can completely revert severe cardiac hypertrophy in models of impaired cardiac pyruvate oxidation (21, 34). Thus, to test the ability of highly enriched medium-chain ketotic feeding to alleviate the cardiac phenotype in the absence of carnitine mediated-fatty acid oxidation, a diet was formulated with octanoate (OctD) as a majority of the diet. Octanoate, in the form of trioctanoin, comprised ∼70% of the total fat and provided ∼74% of the total dietary kilocalories (Fig. 1C). Control and *Cpt2*^*M−/−*^ male and female mice were placed on either OctD or matched control diet (CD) at 3 weeks of age when neither cardiac hypertrophy nor heart failure is detectable in *Cpt2*^*M−/−*^ mice (23). Dietary intervention lasted a total of 4 weeks. While *Cpt2*^*M−/−*^ mice are mildly ketotic on chow diet, as previously reported (23), the octanoate diet induced ketosis in both control and *Cpt2*^*M−/−*^ mice (Fig. 1D). The octanoate diet neither altered body weight, by genotype, nor relative to the CD-fed mice (Fig. 1E) but did reduce adipose depot mass in both genotypes (Fig. 1F). Although not reaching significance, we observed a trend toward slightly reduced heart mass in the OctD-fed *Cpt2*^*M−/−*^ mice compared with CD-fed *Cpt2*^*M−/−*^ mice (Fig. 1G). Echocardiographic analysis confirmed that cardiac hypertrophy in *Cpt2*^*M−/−*^ mice was not attenuated by OctD as indicated by sustained elevated left ventricular mass (Fig. 2A).

We previously reported that *Cpt2*^*M−/−*^ mice had compromised cardiac function (23). Here, *Cpt2*^*M−/−*^ mice fed either chow or OctD presented with increased end-diastolic volume along with a 25% reduction in flow velocity as indicated by Doppler analysis (Fig. 2B, C). Ejection fraction remained significantly lower in *Cpt2*^*M−/−*^ mice independent of diet (Fig. 2C). In *Cpt2*^*M−/−*^ mice, the OctD slightly increased peak-systolic volume, whereas in control mice, the OctD increased end-diastolic volume, stroke volume, and peak-systolic volume by ∼50% (Fig. 2D). Interestingly, while cardiac output was not changed in *Cpt2*^*M−/−*^ mice with OctD, control mice had a ∼1.5-fold increased output (Fig. 2D). Together, these data demonstrate that the octanoate-rich diet modulates cardiac function of control mice, an effect that *Cpt2*^*M−/−*^ mice are impervious to, and that octanoate diet fails to alleviate cardiac hypertrophy or to improve cardiac function in CPT2-deficient hearts.

### Alternative dietary fuels regulate cardiac hypertrophy-related genes

**Fig. 3:**
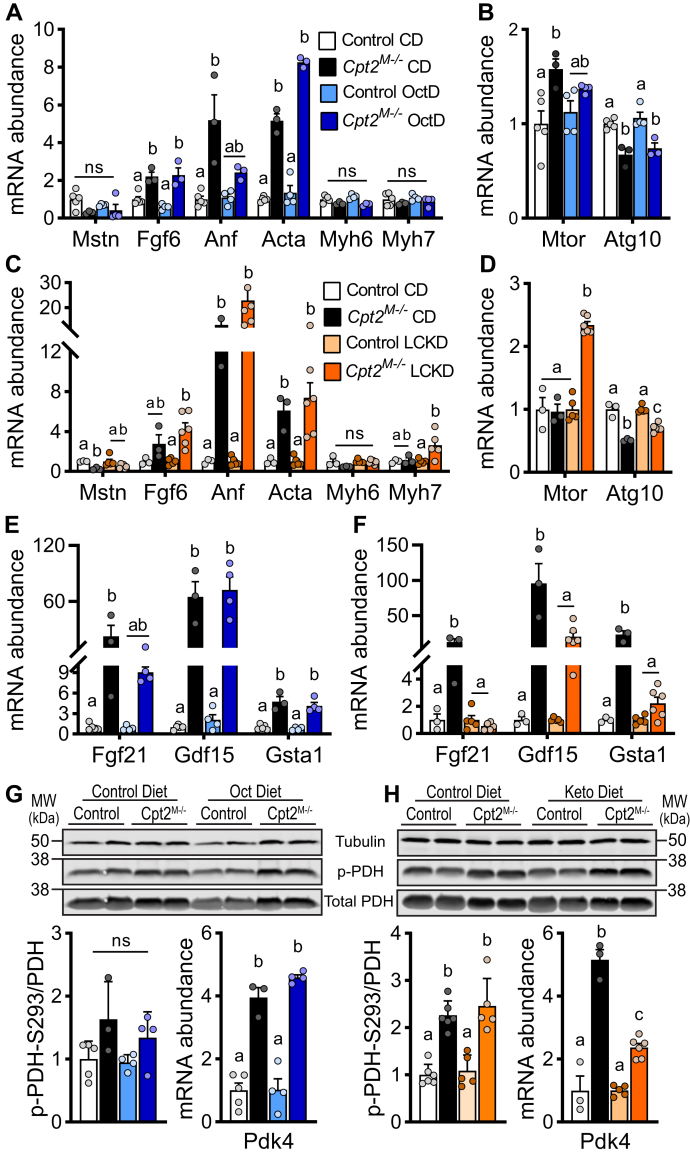
Effects of long- and medium-chain ketogenic diets on cardiac remodeling and metabolic programs. A–F: Cardiac mRNA abundance normalized to control group on control diet (CD) of pathological hypertrophy markers, mTOR pathway, and myokines from control and *Cpt2*^*M−/−*^ mice fed either CD, OctD, or LCKD, females, n = 4–6. G, H: Phosphorylation levels of PDH enzyme at Serine293 and mRNA abundance of PDH kinase (*Pdk4*) as regulators of pyruvate metabolism in hearts from control and *Cpt2*^*M−/−*^ mice fed either CD, OctD, or LCKD, females, n = 4–6. Statistical analysis by 2-way ANOVA. Means depicting a different letter indicate significant differences between groups (*P* ≤ 0.05). Cpt2, carnitine palmitoyltransferase 2; LCKD, long-chain fatty acid ketogenic diet; mTOR, mechanistic target of rapamycin; OctD, octanoate diet; PDH, pyruvate dehydrogenase; Pdk4, pyruvate dehydrogenase kinase 4.

Next, we assessed the molecular response of *Cpt2*^*M−/−*^ hearts to octanoate dietary intervention. OctD modestly reduced cardiac expression of atrial natriuretic factor and increased actin alpha 1 expression in *Cpt2*^*M−/−*^ mice, thus inducing minimal changes on molecular indicators of pathological remodeling compared with CD-fed *Cpt2*^*M−/−*^ mice (Fig. 3A). OctD did not trigger expression of hypertrophy genes in control mice (Fig. 3A, B). The mechanistic target of rapamycin (mTOR) growth pathway is a critical regulator of cardiac hypertrophy (13, 35, 36). While *Cpt2*^*M−/−*^ hearts had increased *mTor,* reduced autophagy marker *Atg10*, and increased phosphorylation of downstream target p70S6K of mTOR, the OctD did not significantly affect these measures for either genotype (Fig. 3B and supplemental Fig. S1A). We previously showed that a ketogenic diet formulated with long-chain fatty acids as the primary fat source (long-chain fatty acid ketogenic diet [LCKD]) did neither alleviate hypertrophy nor prevented progression to heart failure in *Cpt2*^*M−/−*^ mice (23) when administered at 6 weeks of age and for a total of 4 weeks. Similarly, others have suggested that the failing heart increases ketone body utilization, and this could act as a potential contributor to failure (37). Here, we found that LCKD-fed *Cpt2*^*M−/−*^ hearts had increased expression of pathological remodeling markers, namely fibroblast growth factor 6, myosin heavy chain beta, and atrial natriuretic factor (Fig. 3C). LCKD significantly increased *mTor* gene expression but did not significantly increase p70S6K phosphorylation in *Cpt2*^*M−/−*^ hearts (Fig. 3D and supplemental Fig. S1B). These data suggest that LCKD, but not OctD, further exacerbates pathological remodeling in *Cpt2*^*M−/−*^ hearts.

In relation to mitochondrial crosstalk with other tissues and organs, myomitokines are predicted to regulate systemic metabolism in response to mitochondrial metabolic dysfunction (38). Both, OctD and LCKD, significantly reduced the gene expression of the myomitokine fibroblast growth factor 21 in *Cpt2*^*M−/−*^ hearts, and LCKD also reduced growth differentiation factor 15 and glutathione-S-transferase alpha-1 (Fig. 3E, F). Thus, both ketogenic diets attenuated mitochondrial metabolic stress-induced myomitokine expression profiles in *Cpt2*^*M−/−*^ hearts, whereas minimal changes were observed in control hearts. Because medium-chain ketogenic diet corrected hypertrophy in a model of impaired pyruvate metabolism (21, 34), we next questioned the effect of the LCKD and OctD diets on regulators of pyruvate oxidation. *Cpt2*^*M−/−*^ hearts had increased pyruvate dehydrogenase kinase 4 mRNA abundance and PDH phosphorylation, and neither LCKD nor OctD altered these regulators of pyruvate oxidative metabolism in either genotype suggesting that the diets may not directly impact pyruvate oxidation (Fig. 3G–J). Together, these data suggest that LCKD, but not OctD, alleviates myomitokine expression but promotes further exacerbation of mTOR-growth pathway and pathological hypertrophy genes in CPT2-deficient hearts. Minimal effects on gene expression were observed in control mice, suggesting that short-term ketogenic diets rich in either long-chain or medium-chain fatty acids do not promote pathological hypertrophy or metabolic stress-induced myomitokine secretion in the normal heart.

### Cardiac acylcarnitine accumulation is not regulated by octanoate diet

**Fig. 4:**
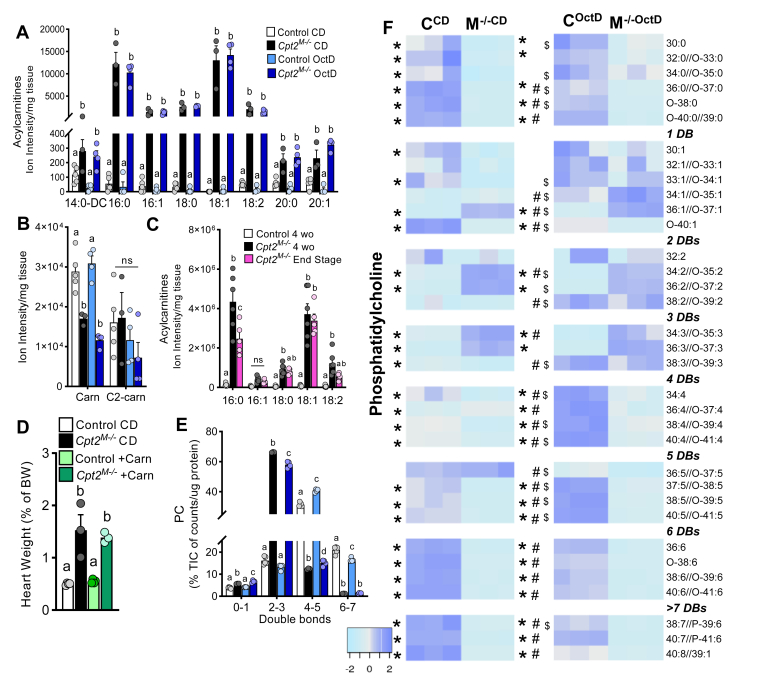
CPT2-deficient hearts are resistant to diet-induced alterations in cardiac acylcarnitines and phospholipid acyl-chain composition. A, B: Cardiac acylcarnitines in control and *Cpt2*^*M−/−*^ mice fed control and OctD, n = 3–5 or (C) at 4 weeks of age and end-stage heart failure, n = 4–6. D: Heart mass in control and *Cpt2*^*M−/−*^ with and without dietary carnitine supplementation, n = 4–5. E: Sum of and (F) heat map for normalized abundance of phosphatidylcholine species arranged by unsaturation degree, in control and Cpt2^M*−*/*−*^ mice fed control or OctD, n = 3. Data are presented as mean ± SEM, all from female mice. Statistical analysis by 2-way ANOVA. Means depicting a different letter indicate significant differences between groups (*P* ≤ 0.05). ∗by genotype, #by diet among controls, and $by diet among *Cpt2*^*M−/−*^. Cpt2, carnitine palmitoyltransferase 2; DBs, double bonds; wo, weeks of.

*Cpt2*^*M−/−*^ hearts accumulate significant amounts of long-chain acylcarnitines (LCACs) (23) on standard diet, so we questioned if the high octanoate diet would alleviate the acylcarnitine accumulation by increasing oxidative flux of carnitine-independent substrates (i.e., ketone bodies and octanoate). Surprisingly, OctD failed to correct the free carnitine deficiency or to attenuate LCAC accumulation in *Cpt2*^*M−/−*^ hearts (Fig. 4A, B). These data suggest that, despite diet-induced surplus of alternative substrates for oxidative metabolism, the steady-state levels of accumulated acylcarnitines in *Cpt2*^*M−/−*^ are not altered by OctD. LCAC accumulation and free carnitine deficiency are suspected regulators of cardiomyocyte function (39, 40, 41, 42). However, in *Cpt2*^*M−/−*^ mice at end-stage disease and near heart failure, total LCACs did not increase, but several species decreased, including palmitoylcarnitine (Fig. 4C). Taken together, these data suggest that *Cpt2*^*M−/−*^ mice on standard low-fat diets reach the maximum capacity of LCAC accumulation that is not reduced by octanoate diet or altered by eminent heart failure. Because the provision of octanoate as alternative fuel did not restore free carnitine levels in *Cpt2*^*M−/−*^ mice, we thought to evaluate the link between carnitine deficiency and cardiac hypertrophy by supplementing mice with free carnitine in the drinking water for 4 weeks, as previously described and validated (25). Free carnitine supplementation failed to rescue cardiac hypertrophy or to modulate gene expression of pyruvate dehydrogenase kinase 4, pathological remodeling markers, mTor, and myomitokines in *Cpt2*^*M−/−*^ mice (Fig. 4D and supplemental Fig. S1C–E). Together, these data suggest that dietary fat, no matter the chain length, and free carnitine supplementation have little impact on cardiac LCAC accumulation in the absence of CPT2.

### CPT2-deficient hearts have reduced highly unsaturated fatty acids in phospholipids and are resistant to octanoate diet-induced membrane desaturation

To determine if LCACs could flux back through the reversible reaction catalyzed by CPT1 into long-chain acyl-CoAs and be incorporated into complex lipids, the acyl chain length and saturation of cardiac membrane lipids were analyzed. Saturated acyl chains, the major substrates for mitochondrial oxidation, were reduced in *Cpt2*^*M−/−*^ heart phospholipids suggesting that acyl chains not used for oxidation are not fluxing into phospholipids. In addition, most of the phosphatidylcholine (PC) species containing ≥4 unsaturated bonds were depleted by 30–90%, whereas those containing -2 or -3 unsaturated bonds were increased by ∼5- and 2-fold, respectively, in *Cpt2*^*M−/−*^ hearts (Figs. 4E, F and supplemental Fig. S2). The OctD elicited changes in control hearts by reducing the membrane content of PCs with -6 or more unsaturated bonds by ∼50% and by increasing PCs of −4 and −5 unsaturated bonds by ∼80% (Figs. 4E, F and supplemental Fig. S2). The *Cpt2*^*M−/−*^ mice, unlike controls, were resistant to diet-induced alterations in phospholipid acyl chain composition (Figs. 4E, F and supplemental Fig. S2). Together, these data demonstrate that loss of CPT2 resulted in an overall reduction in highly unsaturated fatty acids within cardiac membrane phospholipids, an outcome that was not altered by the high octanoate diet, whereas control mice reduced membrane unsaturation in response to the high octanoate diet.

### Free octanoate is an oxidative substrate in liver but not in heart or skeletal muscle

**Fig. 5:**
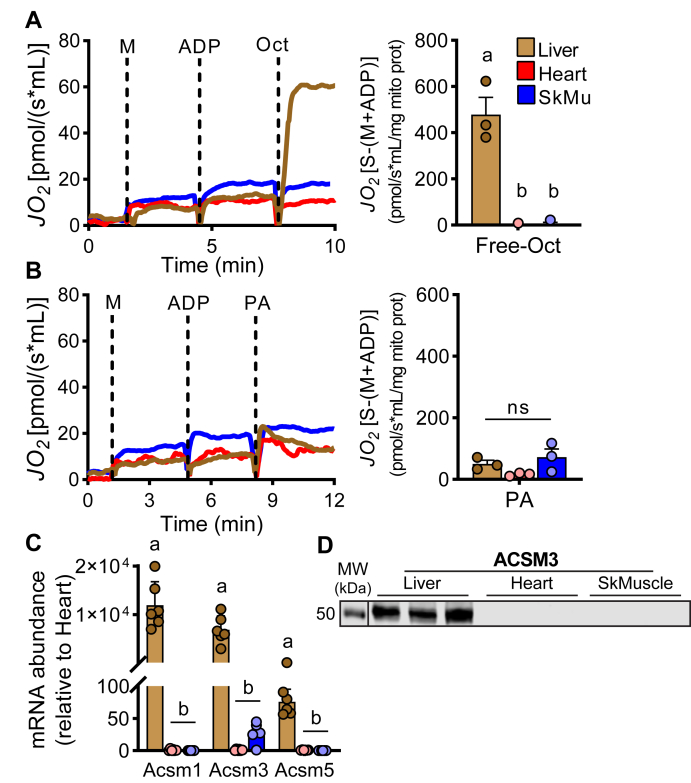
Differential octanoate oxidation between liver, heart, and skeletal muscle. A, B: Rates of oxygen consumption in isolated mitochondria of liver, heart, and skeletal muscle as representative traces over time and quantification of maximum rates during administration of malate (M) and ADP along with free octanoate (Oct) or free palmitate (PA) as substrate, n = 3. Relative abundance of ACSM isoforms by (C) mRNA and (D) expression of ACSM3 protein in liver, heart, and skeletal muscle, n = 3–6. Data are presented as mean ± SEM, all from male mice. Statistical analysis by 1-way ANOVA. Means depicting a different letter indicate significant differences between groups (*P* ≤ 0.05). ACSM, medium-chain acyl-CoA synthetase.

Given the inability of the OctD to rescue *Cpt2*^*M−/−*^ cardiomyopathy, we reasoned that this could relate to intrinsic deficiencies in octanoate uptake, activation, and/or oxidation in cardiac mitochondria. Therefore, oxidative capacity of octanoate between organs was determined in whole-tissue homogenates and isolated mitochondria from liver, heart, and skeletal muscle. For these experiments, the substrate of interest was added along with malate, to support flux through the tricarboxylic acid cycle, and with ADP, to stimulate respiration. In line with the notion that medium-chain fatty acids are oxidized independent of the carnitine shuttle, liver homogenates and isolated mitochondria were fully capable of oxidizing free octanoate in the absence of carnitine (Fig. 5A and supplemental Fig. S3). On the contrary, cardiac and skeletal muscle homogenates and isolated mitochondria showed no appreciable oxidation rates in the presence of free octanoate (Fig. 5A and supplemental Fig. S3). None of the tissues could oxidize the long-chain fatty acid palmitate in the absence of carnitine (Fig. 5B and supplemental Fig. S3) showing that the differential oxidative capacity between liver, heart, and skeletal muscle is chain length dependent. We next questioned why the liver, but not the heart or skeletal muscle, could oxidize free octanoate. Octanoate oxidization through the beta-oxidation spiral is initiated by the matrix enzyme MCAD whose substrate is octanoyl-CoA within the mitochondrial matrix. Octanoyl-CoA can be the product of either CPT2, utilizing CoA and octanoylcarnitine as substrate, or mitochondrial ACSMs, utilizing CoA and free octanoate as substrate. The liver, but not the heart or skeletal muscle, highly expressed several *Acsm* genes, namely *Acsm1*, *Acsm3*, and *Acsm5* (Fig. 5C), as we have previously reported (43). Expression of ACSM3 protein was confirmed in liver mitochondria but could not be detected in skeletal muscle or heart (Fig. 5D and supplemental Fig. S3). Thus, the liver may have the unique ability to activate free octanoate for betaoxidation via ACSM. These data demonstrate that liver mitochondria, but not muscle or heart, can oxidize free octanoate.

### Octanoylcarnitine is oxidized by liver, heart, and muscle in a CPT2-independent manner

**Fig. 6:**
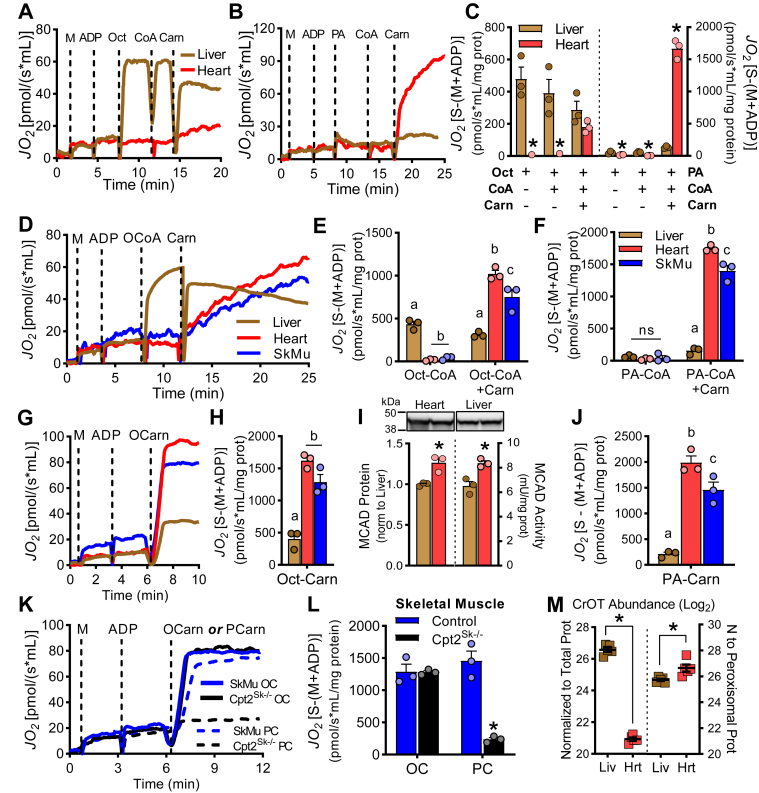
Octanoylcarnitine is oxidized by liver, heart, and muscle in a CPT2-independent manner. A, B: Rates of oxygen consumption in isolated mitochondria of liver and heart as representative trace over time and (C) quantitation of maximum rates during administration of malate (M) and ADP with octanoate (Oct) or palmitate (PA) as substrates, followed by CoA and free l-carnitine (Carn), n = 3. D: Rates of oxygen consumption in isolated mitochondria from liver, heart, and skeletal muscle as representative trace and (E, F) quantitation of maximum rates when given M and ADP with octanoyl-CoA (OCoA) or palmitoyl-CoA followed by carnitine, n = 3. G: Rates of oxygen consumption in isolated mitochondria of liver, heart, and skeletal muscle as representative trace and (H) quantitation of maximum rates given octanoylcarnitine (OCarn) as substrate, n = 3. I:MCAD protein and activity in heart and liver, n = 3. J: Rates of palmitoylcarnitine oxidation in isolated mitochondria of liver, heart, and skeletal muscle. K: Representative trace of oxygen consumption and (L) quantification of maximum rates in isolated mitochondria of skeletal muscle from control or Cpt2^Sk−/−^ mice given octanoylcarnitine and palmitoylcarnitine (PCarn), n = 3. M: Relative carnitine O-octanoyltransferase (CrOT) abundance in isolated mitochondria from liver and heart as detected by discovery proteomics, n = 5. Data are presented as mean ± SEM, all from male mice. ∗*P* ≤ 0.05 by one-way ANOVA (E, F, H, J, and M) or Student's *t*-test (I and J). Means depicting a different letter indicate significant differences between groups (*P* ≤ 0.05). MCAD, medium-chain acyl-CoA dehydrogenase.

We next assessed the ability of the tissues to use the acylcarnitine shuttle for oxidation of octanoate. Therefore, to represent the sequential substrates needed for the acylcarnitine shuttle reactions, free octanoate was used as the oxidative substrate, then CoA was added followed by free carnitine addition. Liver-isolated mitochondria and homogenates showed high rates of oxidation for free octanoate that were not further increased by addition of CoA or carnitine (Fig. 6A, C and supplemental Fig. S3). Conversely, heart mitochondria and homogenates only oxidized free octanoate after both, CoA and carnitine, were added to the reaction buffer (Fig. 6A, C and supplemental Fig. S3). To confirm that the lack of exogenous ATP in our assay conditions was not limiting the matrix ATP supply for ACSM-mediated reactions, an ATP-supplemented buffer was used and again showed high rates of free octanoate oxidation in liver, but not in heart (supplemental Fig. S3D–G). Control experiments were performed using palmitate as substrate to show that liver and heart failed to oxidize free palmitate until both CoA and carnitine were added in agreement with the dependence of long-chain fatty acids on the acylcarnitine shuttle for oxidation (Fig. 6B, C and supplemental Fig. S3). To assess if the lack of ACSMs in the heart limited octanoate oxidation, octanoyl-CoA, the product of ACSM action and substrate for MCAD, was provided as substrate. In liver, rates of octanoyl-CoA oxidation were high and nearly identical to those of free octanoate; in heart, however, octanoyl-CoA was not oxidized (Fig. 6D, E). To determine if octanoyl-CoA was not used by heart because of inability to be transported into the mitochondria, carnitine was added after octanoyl-CoA to allow production of octanoylcarnitine by CPT1 for transport into the mitochondria. Carnitine addition after octanoyl-CoA initiated a slow but steady increase in oxidation rates in the heart (Fig. 6D, E) suggesting that octanoyl-CoA is a viable substrate for the heart but only in the presence of free carnitine. We next questioned if the metabolic signature of skeletal muscle was similar to that of heart, and indeed skeletal muscle oxidation rates closely mimicked those observed in heart (Fig. 6D, E). Control experiments using palmitoyl-CoA followed by free carnitine addition confirmed that liver, heart, and skeletal muscle oxidize long-chain fatty acids only in the presence of carnitine (Fig. 6F). As for maximum respiration capacity, heart and skeletal muscle mitochondria showed appreciably higher oxidative rates for both octanoate and palmitate when CoA and carnitine were subsequently added, compared with liver (Fig. 6E, F). To test respiratory rates when carnitine esters are provided as fuel, octanoylcarnitine was administered to show ∼4-fold greater oxidative rates in muscles compared with liver (Fig. 6G, H). In agreement, the heart presents higher protein abundance and has higher enzymatic activity for MCAD, a mitochondrial matrix dehydrogenase that could potentially set the rate for medium-chain fatty acid beta-oxidation (Fig. 6I). When considering long-chain fatty acid oxidation of palmitoylcarnitine, the heart and muscle had ∼9- and 7-fold greater rates than liver, respectively (Fig. 6J), suggesting that muscle tissues have overall high mFAO capacity.

**Fig. 7:**
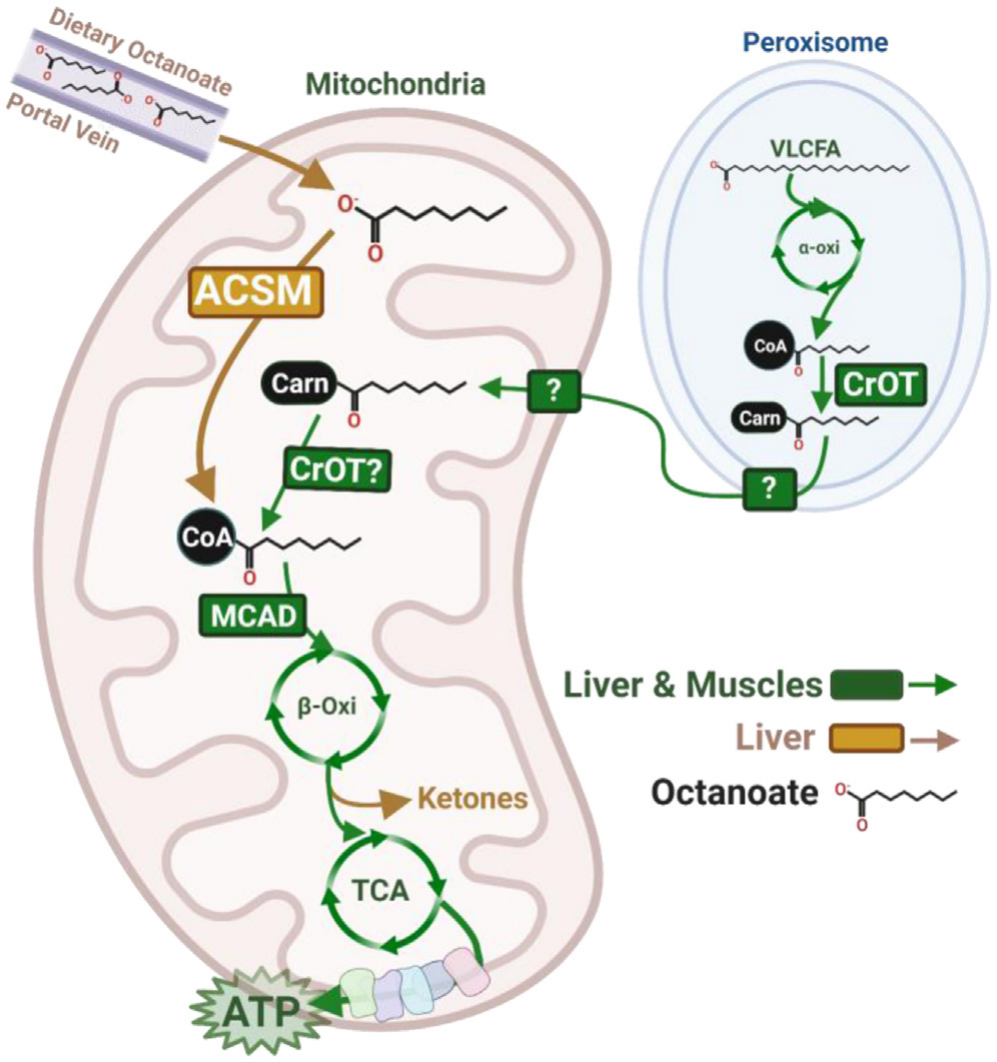
Schematic of octanoate oxidation in liver and muscles. Speculative metabolic pathway for octanoate oxidation in liver and muscles (cardiac and skeletal). Dietary-free octanoate reaches the liver through the portal circulation where liver, but not muscle, can activate free octanoate within the mitochondria via medium-chain acyl-CoA synthetases (ACSM). Muscles and liver mitochondria metabolize octanoylcarnitine as a product of peroxisomal oxidation. The enzyme that converts octanoylcarnitine back to octanoyl-CoA in the mitochondria is not CPT2 but could be carnitine O-octanoyltransferase (CrOT). Octanoyl-CoA enters mitochondrial oxidation through medium-chain acyl-CoA dehydrogenase (MCAD) and can be oxidized for energy production in liver and muscles or can be used for ketone body production in liver. β-Oxi, beta-oxidation; Carn, free carnitine; CoA, coenzyme A; TCA, tricarboxylic acid cycle; VLCFA, very long chain fatty acid. Created with BioRender.com.

Because heart and skeletal muscle had higher oxidation rates for octanoyl-CoA + Carn and octanoylcarnitine, and because the only known mitochondrial acyl-CoA-acylcarnitine exchanger is the carnitine shuttle (CPT1-CACT-CPT2), we decided to determine if carnitine-palmitoyltransferase activity is required for oxidation of octanoylcarnitine. Oxidation rates were determined in skeletal muscle mitochondria from control and skeletal muscle-specific CPT2-deficient mice (*Cpt2*^*Sk*−/−^) (24). In agreement with our previous findings in *Cpt2*^*M−/−*^ hearts (23), the oxidation rates of octanoylcarnitine in skeletal muscle mitochondria were not different between control and *Cpt2*^*Sk*−/−^ (Fig. 6K, L). Confirmation of CPT2 deficiency was evident by the nearly absent rates of palmitoylcarnitine oxidation in *Cpt2*^*Sk*−/−^ muscle (Fig. 6K, L). Next, we assessed the involvement of CrOT, a peroxisomal enzyme that performs the same reaction as CPT2 but has very high preference for octanoyl-CoA as substrate. We detected CrOT in mitochondrial fractions from both liver and heart, with higher abundance in liver when normalized to total proteins discovered (Fig. 6M, left panel). When correcting CrOT enrichment relative to peroxisomal proteins, CrOT was found at similar levels in the liver and heart (Fig. 6M, right panel). While it is not clear if CrOT is present in the mitochondria, these data suggest the possibility of CrOT activity replacing CPT2 for matrix octanoyl-CoA formation and subsequent oxidative metabolism. All together, these data suggest that the liver, heart, and muscle comprise the necessary machinery to oxidize octanoate in a carnitine-dependent, but CPT2-independent, manner, and that liver is capable of CoA- and carnitine-independent octanoate oxidation (Fig. 7).

## Discussion

We have previously reported that mediators of cardiac hypertrophy in *Cpt2*^*M−/−*^ mice are distinct from that of other common models of hypertrophy (23). Specifically, we have shown that unlike hypertension-induced and ischemia-induced models (36, 44, 45, 46, 47), the cardiac hypertrophy triggered by CPT2 deficiency is resistant to attenuation by rapamycin, an mTor inhibitor, and by trichostatin A, a deacetylase inhibitor (23). Because both rapamycin and trichostatin A improve mitochondrial oxidative metabolism in normal hearts (48, 49, 50, 51, 52, 53), our data suggested that restoration of cardiac fatty acid oxidation is required for the effectiveness of these therapies. In agreement, both high-fat diet and medium-chain ketogenic diet were shown to improve severe cardiac hypertrophy in models of impaired cardiac pyruvate oxidation (21, 34). These results suggest that increased flux of either ketones or fatty acids through oxidative metabolism can compensate for the loss of carbohydrate-oxidative metabolism and restore cardiac structure. Ketogenic diets have also been documented to paradoxically reduce ketolytic activity in the heart and increase long-chain fatty acid oxidative metabolism (54). Our data herein unequivocally show that in the absence of long-chain fatty acid oxidative flux in the mitochondria, medium-chain fatty acid diets or ketogenic diets cannot rescue cardiac hypertrophy. Thus, these combined data suggest that the ability of ketogenic diets to increase carnitine-dependent fatty acid oxidation in the heart is facilitating cardioprotective effects and that without long-chain fatty acid oxidative metabolism, cardioprotection cannot occur. This is surprising considering that medium-chain oil therapy is a standard of care for patients with long-chain fatty acid oxidation disorders (55, 56).

The ability of medium-chain fatty acids of eight carbons or less to produce ketone bodies following an oral bolus suggests that these fatty acids are preferred substrates for liver metabolism. Indeed, medium-chain fatty acids are well known to be digestively absorbed into the portal vein for direct transit to the liver. Thus, medium-chain fatty acids bypass absorptive processing of common dietary fats (i.e., long-chain fatty acids), which are packaged into lipoprotein particles by enterocytes and released into the lymphatics, which merge into the vascular system near the heart. Thereby, while dietary long-chain fatty acids are first presented to the heart and then travel throughout the body passing through skeletal muscle before gaining access to the liver, dietary medium-chain fatty acids are directly routed from the gut to the liver. The liver is rich in the enzymatic machinery required for medium-chain fatty acid oxidation and is even capable of oxidizing free octanoate. Hepatocytes also incorporate octanoate into triglycerides for secretion into the circulation within lipoprotein particles (57). Therefore, heart and muscle may access majority of dietary octanoate in the form of complex lipids within lipoproteins. It is likely that high octanoate from the diet is predominantly processed by the liver for energy production and ketogenesis; and that the heart and skeletal muscles do not have direct access to dietary free medium-chain fatty acids and therefore do not express the required metabolic machinery to oxidize free octanoate. Muscles may predominantly be exposed to octanoate in the form of octanoylcarnitine, as a product of peroxisomal oxidation and peroxisomal CrOT enzyme action. Thus, mammals may be biologically adapted to metabolize free octanoate and octanoylcarnitine in liver but only octanoylcarnitine in muscles.

To further delineate the mechanisms by which liver oxidizes free octanoate but cardiac and skeletal muscles cannot, we considered the fact that octanoate must be converted into octanoyl-CoA within the mitochondrial matrix for subsequent oxidative metabolism. Acyl-CoA moieties can be formed in the matrix by the transferase action of CPT2 when acylcarnitine and CoA are substrates or by the ligation action of ACSMs when free fatty acids and CoA are provided. Here, we demonstrate a striking distinction between liver and muscles for mitochondrial expression of ACSMs. The liver has high abundance of three of the five known ACSMs while ACSM expression is nearly absent in the heart and skeletal muscle (43). The action of these ASCMs may equip the liver with a unique advantage to activate free octanoate within the mitochondrial matrix for oxidative metabolism. Thus, the long-standing notion that medium-chain fatty acids are oxidized independent of carnitine stands true for the liver, and possibly for other tissues that express ACSMs such as kidney and gut (43), but not for muscles where carnitine-independent octanoate oxidation is not a viable mechanism.

Acylcarnitines, short to long, cannot freely cross membranes, unlike their free fatty acid counterparts (58). Therefore, acylcarnitines are transported between cellular compartments, and in and out of cells, through protein-mediated processes. CACT is the best characterized example of transporter of LCACs, located in the inner mitochondrial membrane. Indeed, patients with defects in CACT have identical clinical presentation to those with CPT2 deficiency (59). While transport of free octanoate into the matrix does not require a transporter, its esters, octanoylcarnitine and octanoyl-CoA, theoretically should require transporters because of their more hydrophilic nature. Intriguingly, CACT-deficient leukocytes cannot oxidize palmitate but are able to oxidize octanoate (60). We also found that CPT2-deficient hearts and muscles are fully capable of oxidizing octanoylcarnitine similar to our results herein using mouse CPT2-deficient muscles. The mitochondrial transporter used to facilitate octanoylcarnitine oxidation remains unestablished. Curiously, we found that liver presented with an additional, potentially transport-mediated, advantage for oxidation of octanoyl-CoA, which was readily oxidized by liver, but not muscles, in a manner independent of carnitine. These data suggest that either liver expresses mitochondrial transporters for octanoyl-CoA itself or that the CoA is cleaved from octanoate prior to entry into the mitochondrial matrix by acyl-CoA thioesterases that are active on medium-chain acyl-CoAs and abundant in liver (43, 61). Because our data for liver octanoyl-CoA oxidation are identical between homogenates and isolated mitochondria, we would argue that matrix thioesterases (62) are not involved, unless the formation of octanoyl-CoA within the matrix is not required for mitochondrial octanoate oxidative metabolism. Given our data showing the rapid rise to identical rates of oxidation in liver for all three forms of octanoate, the free, -CoA, and -carnitine esters, we suggest that the transport-mediated processes and/or enzymatic metabolism of octanoate and its metabolites occur in a rapid and unrestrictive fashion in liver. Previous work demonstrated that inhibition of CPT1 with etomoxir or malonyl-CoA in muscle mitochondria does not effect octanoylcarnitine oxidation, suggesting that octanoylcarnitine oxidation occurs independent of CPT1 in muscle (63). Importantly, our findings using a CPT2 deficiency model show that CPT2 is not involved in the oxidation of octanoylcarnitine in muscles, yet the transporter and transferase responsible for octanoylcarnitine transport into the mitochondrial matrix and for its oxidative metabolism remain undefined. A possibility is the presence of CrOT or a CrOT-like enzyme in the mitochondrial matrix where it could convert octanoylcarnitine to octanoyl-CoA. Indeed, we identified CrOT protein in mitochondrial preparations of both liver and heart, thus there is a potential for CrOT to contribute to octanoylcarnitine oxidation in both tissues; however, its localization to the matrix has not been established.

It is not clear the exact chemical form and location that acylcarnitines take when accumulating inside cells. The amphipathic nature of acylcarnitines could hypothetically allow accumulation as micellular structures and/or be integrated into the amphipathic layers within membranes. Membrane accumulation of LCACs could increase packing defects, causing increased space between phospholipids. Packing defects are also caused by excessive changes to the ratio of saturated:unsaturated fatty acids within membranes (64, 65). This biophysical property is important for membrane curvature and protein-membrane interactions. It is possible that the reduction in highly unsaturated phospholipid species in *Cpt2*^*M−/−*^ hearts, and in *Cpt2*^*Sk−/−*^ muscles (24), is a compensatory mechanism to maintain membrane dynamics because of membrane acylcarnitine integration. Reduced membrane unsaturation is also reported in individuals with genetic defects in mFAO, and these patients are often supplemented with dietary DHA (66).

The OctD affected cardiac function of control mice, resulting in increased cardiac output. The increase in cardiac output could be the result of increased peripheral blood perfusion demands in response to increased oxygen requirement for high rates of oxidative metabolism during ketotic dietary conditions. However, the *Cpt2*^*M−/−*^ mice were impervious to these diet-induced effects and maintained similar cardiac structural dimensions and functional outputs between the two diets indicating that metabolic remodeling triggered by CPT2 deficiency occurs prior to dietary manipulation and was not further changed by octanoate provision. Increasing the fatty acid chain length by one increment to decanoic acid necessitates the requirement of the carnitine shuttle for oxidation (67); therefore, patients with defects in long-chain mFAO should be supplemented with carefully curated medium-chain oils containing eight carbons or lower (68). Our data also suggest that while the liver does not require carnitine for octanoate oxidation, muscles do require carnitine. Because patients with fatty acid oxidation disorders present with free carnitine deficiency, the use of free carnitine supplementation is sometimes implemented; however, carnitine supplementation remains debated because of concerns that it facilitates cardiac LCAC accumulation and subsequent cardiac arrhythmias (66). Our data herein suggest that free carnitine supplementation along with medium-chain therapy could facilitate the oxidation of medium-chain fatty acid oxidation in heart and muscle tissues.

Together, our findings demonstrate that liver mitochondria have the ability to oxidize free octanoate and octanoyl-CoA in the absence of carnitine, whereas muscle and heart mitochondria do not. However, liver, heart, and muscles oxidize octanoylcarnitine, independent of CPT2. We speculate that this differential oxidative capacity is a biological adaptation to the manner in which octanoate and its metabolites are presented to the mitochondria within these tissues. Specifically, the liver could be predominantly exposed to the free form of octanoate from a dietary source during intestinal absorption, as well as, from peroxisomal oxidation, whereas muscles are more exclusively exposed to octanoylcarnitine as a product of peroxisomal oxidation. Our work also suggests that, as part of such biological adaptations, the liver and heart express distinct enzymatic machinery for octanoate oxidation, such as ACSMs (Fig. 7). Finally, failure of octanoate ketogenic diet to restore normal cardiac structure and function suggests that oxidation of long-chain fatty acids is critical and irreplaceable for cardiac health.

## Data availability

All the data are contained within the article.

## Supplemental data

This article contains supplemental data.

## Conflict of interest

The authors declare that they have no conflicts of interest with the contents of this article.
