# Supplementary material for: Octanoate is differentially metabolized in liver and muscle and fails to rescue cardiomyopathy in CPT2 deficiency
Source: J Lipid Res. 2021 Mar 20;62:100069. doi: 10.1016/j.jlr.2021.100069 (PMC8082564; doi:10.1016/j.jlr.2021.100069)
Supplement: Supplemental Table S1 and Figures S1 to S3 [file mmc1.pdf]

Figure S1. Free carnitine supplementation did not alter metabolic or hypertrophy transcriptional signature.

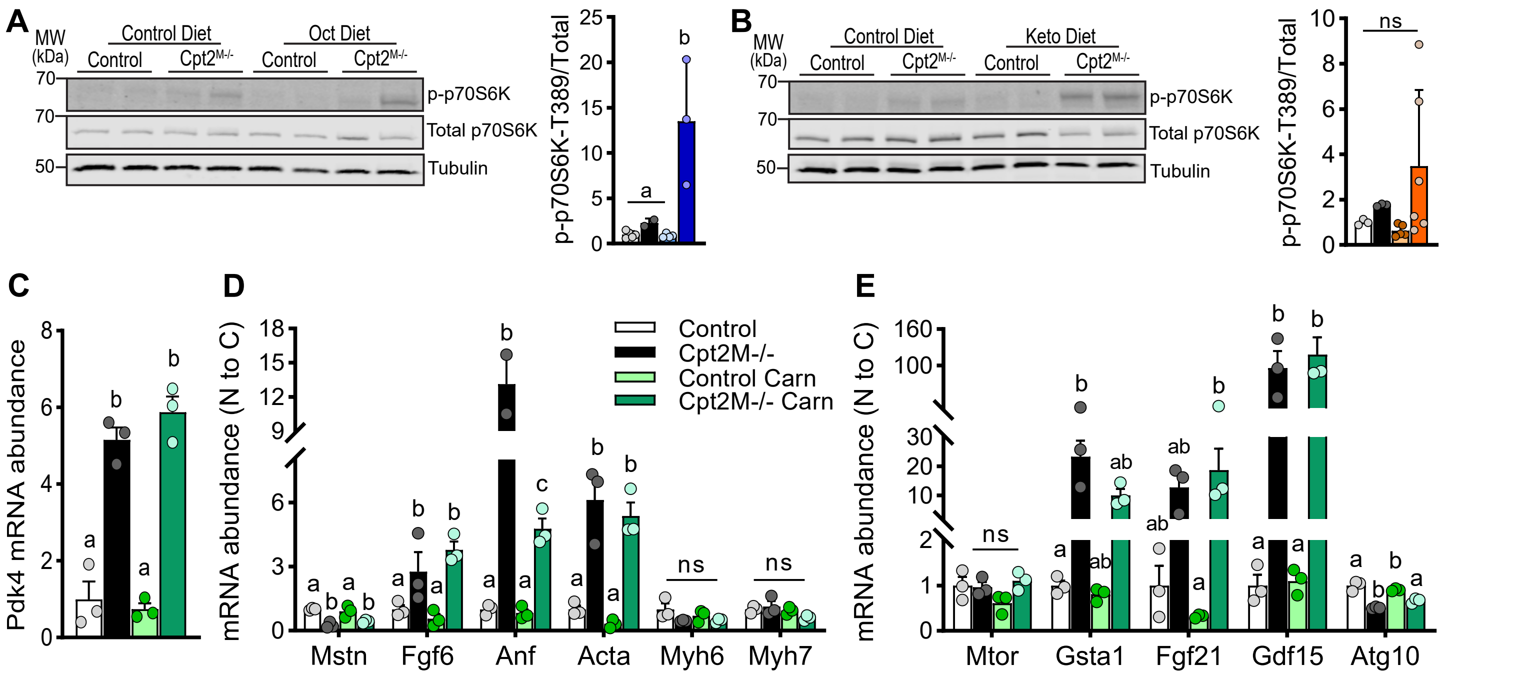

Figure S1. Free carnitine supplementation did not alter metabolic or hypertrophic transcriptional signature. Cardiac p70S6K phosphorylation levels relative to total in control and Cpt2M-/- mice fed either (A) octanoate-enriched diet (OctDiet) or (B) standard ketogenic diet (KD), n=3-6. (C) mRNA abundance of genes regulating pyruvate (oxidation, (D) pathologycal cardiac remodeling, and (E) mTor-myokines in control and Cpt2M-/- with and without dietary carnitine supplementation, n=4-5. Data presented as mean±SEM, all from female mice. Statistical analysis fby 2-way ANOVA. Means depicting a different letter indicate significant differences between groups (P≤0.05).

Figure S2: CPT2 deficient hearts are resistant to diet-induced alterations in cardiac phospholipid acyl-chain composition

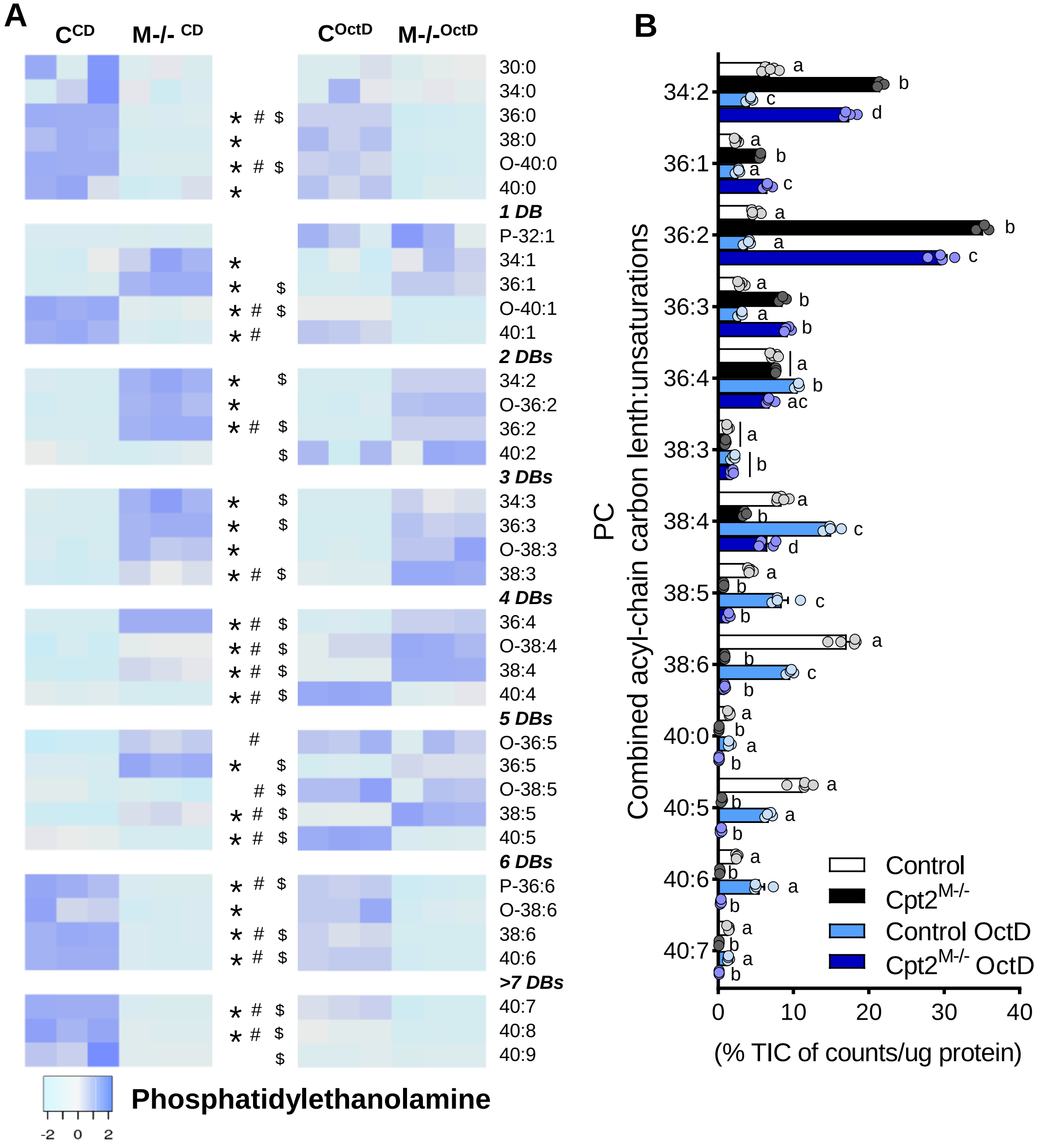

Figure S2: CPT2 deficient hearts are resistant to diet-induced alterations in cardiac phospholipid acyl-chain composition. (A) Heatmap for normalized abundance of phosphatidylethanolamine species and (B) high abundant (above 2% of total) phosphatidylcholine species arranged by combined acyl-chain length and unsaturation degree, in control and Cpt2$^{M-/-}$ mice fed control or OctD, n=4-5. Data presented as mean±SEM, all from female mice. Statistical analysis by 2-way ANOVA. Means depicting a different letter indicate significant differences between groups (P<0.05). * by genotype, # by diet among controls, and $ by diet among Cpt2$^{M-/-}$ for heatmap.

Figure S3. Differential octanoate oxidation between liver and heart homogenates.

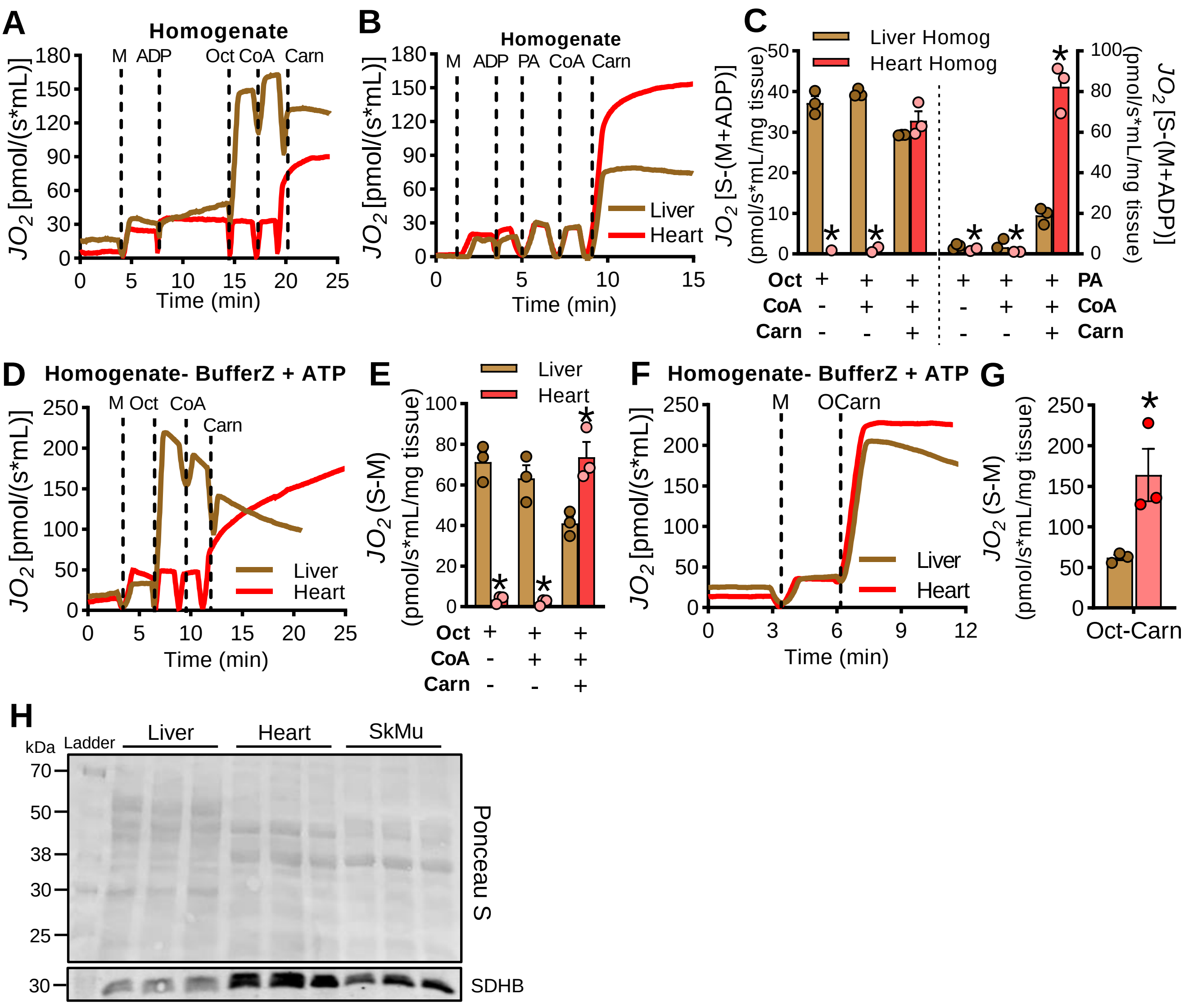

Figure S3: Differential octanoate oxidation between liver and heart homogenates. (A, B) Oxygen consumption in homogenate of liver and heart as representative traces over time and (C) quantitation of maximum rates during administration of malate (M), ADP, free octanoate (Oct) or free palmitate (PA) as substrate, followed by CoenzymeA (CoA) and free L-carnitine (Carn); n=3. (D, F) Oxygen consumption in homogenate of liver and heart incubated in a buffer containing ATP as representative traces over time and (E, G) quantitation of maximum rates during administration of malate (M), free octanoate (Oct) or Octanoyl-carnitine (OCarn) as substrate, followed by CoenzymeA (CoA) and free L-carnitine (Carn); n=3. (H) Total protein by Ponceau S staining on Western Blot from liver, heart and skeletal muscle isolated mitochondria (top) and fraction enrichment and loading control by targeting Succinate Dehydrogenase B (SDHB) (bottom), n=3. Data presented as mean±SEM, all from male mice. * p≤0.05 compared to liver by Student's t-test.

Supplemental Table 1. Composition of octanoate-enriched, long chain ketogenic and matched-control diets.

	Control Diet	Octanoate Diet	LC Keto Diet
Source<br>Catalog Number	Envigo<br>TD 94045	Envigo<br>TD 170585	Bioserv<br>F6689
		**g/kg**	
Casein	200	300	165
L-Cystine	3	3	-
Corn Starch	397.5	-	-
Maltodextrin	132	65	-
Cellulose	50	145.42	85.82
Trioctanoin	-	305	-
Lard	-	110	401.74
Safflower Oil	-	15	-
Soybean Oil	70	-	-
Corn Oil	-	-	96.43
Anhydrous Milkfat	-	-	168.73
Mineral Mix with Ca & P	-	18.5	-
Mineral Mix AIN-93-MX	35	-	-
Mineral Mix AIN-76	-	-	23.7
Calcium Phosphate, Dibasic	-	8.5	25.82
Calcium Phosphate, Monobasic	-	-	9.06
Calcium Carbonate	-	10.75	-
Vitamin Mix AIN-93-VX	10	15	-
Vitamin Mix AIN-76A			17.7
Choline Bitartrate	2.5	3.75	3.5
DL-Methionine	-	-	2.5
Ethoxyquin	-	0.08	-
TBHQ	0.014	-	-
		**% by weight**	
Protein	17.7	26.4	15.0
Carbohydrate	60.1	7.6	1.7
Fat	7.2	43.3	66.7
